# Evidence that microRNAs are part of the molecular toolkit regulating adult reproductive diapause in the mosquito, *Culex pipiens*

**DOI:** 10.1371/journal.pone.0203015

**Published:** 2018-11-29

**Authors:** Megan E. Meuti, Robin Bautista-Jimenez, Julie A. Reynolds

**Affiliations:** 1 Department of Entomology, The Ohio State University, Columbus, Ohio, United States of America; 2 Department of Evolution, Ecology, and Organismal Biology, The Ohio State University, Columbus, Ohio, United States of America; University of Nevada Reno, UNITED STATES

## Abstract

For many insects, diapause is the primary mechanism for surviving unfavorable seasons. Some aspects of diapause regulation are well known, but we still lack a mechanistic understanding of molecular mechanisms that control the diapause pathway. Accumulating evidence suggests microRNAs regulate diapause in evolutionarily diverse insect species including flesh flies and moths, and, it is likely that microRNAs regulate multiple characteristics of diapause, including arrested egg follicle development and fat hypertrophy, in females of the Northern house mosquito, *Culex pipiens*. To investigate microRNA regulation of diapause in this species, we measured changes in egg follicle development and total lipid content over 22 days following adult emergence. We also evaluated changes in the abundance of candidate microRNAs associated with these physical changes during the same time frame. We found egg follicle size and lipid content were nearly the same in diapausing and nondiapausing females on the day of adult emergence, and then diverged over time such that by day 22 diapausing females had significantly smaller egg follicles and higher total lipids than their nondiapausing counterparts. Several microRNAs associated with lipid metabolism in insects, including miR-14-3p, miR-277-3p, and miR-305-5p, were underexpressed in diapausing females compared to nondiapausing females on the day of adult emergence, which suggests microRNA regulation occurs ahead of observed changes in these two features of the diapause phenotype. We also found miR-309-3p, miR-375-3p which stimulate ovarian development in other mosquito species, were underexpressed in diapausing females of *Cx*. *pipiens* at times after diapause is fully established and may be responsible for the arrest in ovarian development in this species. Taken together, our results demonstrate that changes in the abundance of some microRNAs is associated with phenotypic changes in diapause *Cx*. *pipiens* and suggests this epigenetic mechanism is part of the molecular toolkit regulating diapause.

## Introduction

Diapause is an alternative developmental pathway that provides insects, and other animals, a means to survive periods of inimical environmental conditions and exploit seasons with abundant resources. Diapause is a dormant state characterized by developmental arrest, metabolic depression, and enhanced tolerance of environmental stresses. This anticipated response is endogenously regulated and induced during a genetically determined stage of the life-cycle in response to token stimuli (e.g. changes in photoperiod, temperature, or food quality) that signal the advent of unfavorable conditions. Perception of the appropriate stimuli promotes changes in gene regulatory networks that ultimately lead to physiological changes that define the diapause phenotype [1]. Ecologically relevant token stimuli that induce the diapause program are known for many insects [2–5], and diapause-relevant changes in gene expression have been documented for a variety of arthropod species [6–13]. Furthermore, many aspects of diapause have been particularly well-characterized in several mosquito species (reviewed in [14, 15]). However, many details of the regulatory networks that mediate the diapause response remain undefined. A particular knowledge gap is how epigenetic processes, defined here as any process that can alter the phenotype independent of changes to the genotype, may regulate diapause induction, maintenance, and/or termination. The goal of the current study was to explore the role of microRNAs (miRNAs), one type of epigenetic mechanism, in regulating reproductive diapause in adult females of the Northern house mosquito, *Culex pipiens*.

MiRNAs are small (18–25 nucleotides) non-coding RNAs that post-transcriptionally regulate gene expression though their interactions with target gene transcripts. Mature miRNA sequences, processed from the 3’ or 5’ arms of a single-stranded hairpin precursor, bind to Argonaut 1 (Ago1) within the RNA Induced Silencing Complex (RISC) and guide the complex to target mRNAs. Once bound to the RISC, miRNAs can increase expression of their targets [16–19]; but more commonly, miRNAs silence target genes via transcript degradation or translation repression. MiRNAs are emerging as regulators of diapause and other dormant states. They have been implicated as regulators of numerous diapause-relevant biological processes including cell-progression [20, 21], developmental timing [22, 23], metabolism [24, 25], and stress-resistance [26–28]. A number of recently published studies have identified miRNAs that are differentially regulated in diapausing insects and other animals including diapausing embryos of the crustacean *Artemia parthenogenetica* [21], the killifish *Austrofundulus limnaeus* [29], and insects including the flesh fly *Sarcophaga bullata* [30] the mosquito *Aedes albopictus* [31], and the moth, *Helicoverpa zea* [32]. Taken together, the accumulating evidence on miRNA function and abundance in diapausing animals suggests that miRNAs are part of the “diapause toolkit” [9] that regulates diapause across species.

The goal of the present study is to evaluate changes in the abundance of evolutionarily conserved, candidate miRNAs in diapausing adults of a *Culex pipiens*, a mosquito that is an established model for studying adult-reproductive diapause. Female adults of this species survive the winter by entering diapause approximately 5 days after adult emergence in response to short day lengths (< 12 h light per 24 h) received during the 4^th^ larval instar, pupal and early adult stages of development [33, 34]. The diapause phenotype in this species is characterized by a lack of host-seeking behavior and arrested ovarian development, which are features that define adult-reproductive diapause [33–36]. Diapause in *Cx*. *pipiens* also includes fat hypertrophy (i.e., accumulated lipid stores), suppressed metabolism, and enhanced resistance to stress from low temperatures, desiccation, and pathogens, which are common changes that define diapause regardless of the stage when diapause occurs [37–40]. In addition, the roles of juvenile hormone and insulin signaling in establishing and maintaining diapause have been well described for this species [41–42], and components of the circadian clock have been implicated as regulators of diapause in this species [43]. This knowledge about the regulation of diapause, as well as information provided about the biological functions of numerous miRNAs in other mosquito species [31, 44–47] makes *Cx*. *pipiens* an ideal model for probing miRNA regulation of adult-reproductive diapause.

In this study we used quantitative reverse-transcript PCR (qRT-PCR) to measure candidate miRNAs that were selected because they have experimentally verified roles in diapause-relevant processes (e.g. circadian clock, ovarian development, insulin signaling, or metabolism) or are known to be differentially regulated in diapausing pupae of the flesh fly, *S*. *bullata* [30]. We measured miRNA abundance in diapausing and nondiapausing adult females 0, 5, 12, and 22 days after adult emergence, providing information about changes in miRNA profiles over the adult lifespan that may be related to reproductive development and aging. We also measured two phenotypic markers of diapause, egg follicle length and fat content, in female mosquitoes at these times. Finally, we measured changes in miRNA abundance in nondiapausing females after they were given a blood meal to evaluate the possible role of miRNAs in regulating processes related to blood-feeding and ovarian development in diapausing and nondiapausing *Cx*. *pipiens*. Taken together, these data provide evidence that changes in microRNA abundance are associated with diapause in female adults of *Cx*. *pipiens* and may be responsible for at least some aspects of the diapause phenotype, namely egg follicle maturation and lipid accumulation, in this species.

## Methods

### Insect rearing

The established laboratory colony of *Cx*. *pipiens* (Buckeye strain) was maintained as previously described [35, 43]. Mosquitoes in the main colony were kept at 25°C, 75% relative humidity under a long-day photoperiod (16 h light: 8 h darkness). Larvae were provided dried fish food (Tetramin; Blacksburg, VA USA). Adults were provided unlimited access to 10% sucrose solution. Chicken blood (Pel-freez Biologicals; Rogers, AR, USA) was provided using an artificial membrane system (Hemotek; Lancashire, UK) approximately 10 days post-adult emergence, and egg rafts were collected approximately 5 days later. To generate diapausing adults, larvae and pupae were held at 18°C, 75% relative humidity under a short-day photoperiod (8 h light: 16 h darkness). Diapausing adults had access to sugar water for the first 10 days of adult life and then sugar water was removed to simulate the lack of food in their natural environment. Nondiapausing adults used in these studies were generated by rearing all life stages under a diapause-averting, long-day photoperiod (L:D 16:8) with constant access to sugar water. Both diapausing and nondiapausing mosquitoes were reared at the same low temperature (18°C) to ensure that diapause status, and not temperature, was the only factor impacting egg follicle development, lipid accumulation and miRNA expression.

### Measurement of ovarian development and total lipid content

The ovaries of 12 individual nondiapausing and diapausing females from each time point (0, 5, 12, and 22 days after adult emergence; n = 96 females total) were dissected in 0.9% saline solution, and the length of 10 egg follicles/female were measured under 200X magnification (Zeiss Axioskop, Thornwood, NY) as previously described [43].

The lipid content was measured in 4–5 diapausing and nondiapausing individual mosquitoes at the same developmental time points (0, 5, 12 and 22 days after adult emergence). In brief, dried mosquitoes were weighed, and the lipid content in each female was obtained using a modified Vanillin assay [48] as previously described [49] and normalized to that female’s dry weight.

### Sampling regime for miRNA expression studies

Changes in miRNA abundance were assessed in diapausing and nondiapausing females that had not been given a blood meal. Females were collected 0, 5, 12, and 22 d after adult emergence. All samples were collected 4 h after lights turned on (ZT4), and 4 biological replicates each containing 4–5 whole body, adult females were collected for each day.

Changes in miRNA abundance related to blood feeding, and possibly to stimulation of ovarian development, were measured in nondiapausing females 12 d after adult emergence. The experimental group was given a blood meal 36 h prior to sampling (i.e. were blood fed 10 d post-emergence). The control group, also sampled 12 d post-emergence, was only given sugar water and had never been given a blood meal. For each time point, there were 4 replicate samples of 4–5 females each. At the time of sampling, female mosquitoes were flash frozen and stored at -80 °C until they were processed.

### Quantitative reverse-transcription PCR

Abundance of candidate miRNAs was measured using qRT-PCR as previously described [30]. Total RNA was isolated from whole-body, female mosquitoes using the mirVana miRNA Isolation kit (ThermoFisher; Waltham, MA, USA) according to the manufacturer’s directions. Reverse transcription of 2 μg of total RNA was carried out using the miScript II RT kit (Qiagen; Valencia, CA, USA) according to the manufacturer’s directions for HiSpec buffer, which is designed to specifically transcribe mature miRNAs. Relative abundance of each candidate miRNA was measured using an iQ5 Multicolor Real-time PCR Detection System (Bio-Rad; Hercules, CA, USA) and miScript Primer Assays (Qiagen) which use one universal primer and one primer designed to detect a specific miRNA sequence. Sequences used for microRNA specific primers had previously been identified in *Cx*. *quinquefasciatus*, a closely related species that does not enter diapause, and were taken from miRBase [50, 51]. Primer performance conformed to MIQE standards for efficiency [52] as shown in S1 Table. Cycling parameters were 94°C for 15 min followed by 40 cycles of 94°C for 15 s, 55°C for 30 s and 72°C for 30 s. Melt curve analysis indicated only one product was formed under these conditions.

Relative miRNA abundance was evaluated in 3–4 replicate samples for each group with three technical replicates for each miRNA assay using a modified 2^-ΔCt^ method [53]. Briefly, background subtracted fluorescence data were exported from the Bio-Rad iQ5 software and were smoothed and normalized as previously described [54]. These normalized data were used to determine the threshold cycle (Ct) for every technical replicate for each sample, and the average technical replicate for each biological sample was calculated. Extensive analysis demonstrated that the abundance of the miRNA let-7 was consistent across all of our samples (S1 Fig). Hence, the Ct values of the biological replicates were averaged and normalized by subtracting the average Ct for let-7 which served as an internal reference. The resulting value was log transformed to give the relative miRNA abundance (2^-ΔCt^).

### Statistical analysis

All statistical analyses were performed in R.3.32 (R Core Team, 2017). Changes in average egg follicle length, lipid content, and miRNA abundance over time (i.e. day 0 to day 22) for a single mosquito type (i.e. diapausing or nondiapausing) were evaluated using a One-way ANOVA followed by Tukey’s post-hoc test. Differences between diapausing and nondiapausing females for each time point tested were evaluated using Student’s t-test, as were differences between sugar fed and blood fed females. To minimize type 1 errors, p-values for Student’s t-test were corrected using the False Discovery Rate (FDR) method [55].

## Results

### Changes in ovarian length and lipid content related to aging and diapause development

**Fig 1:**
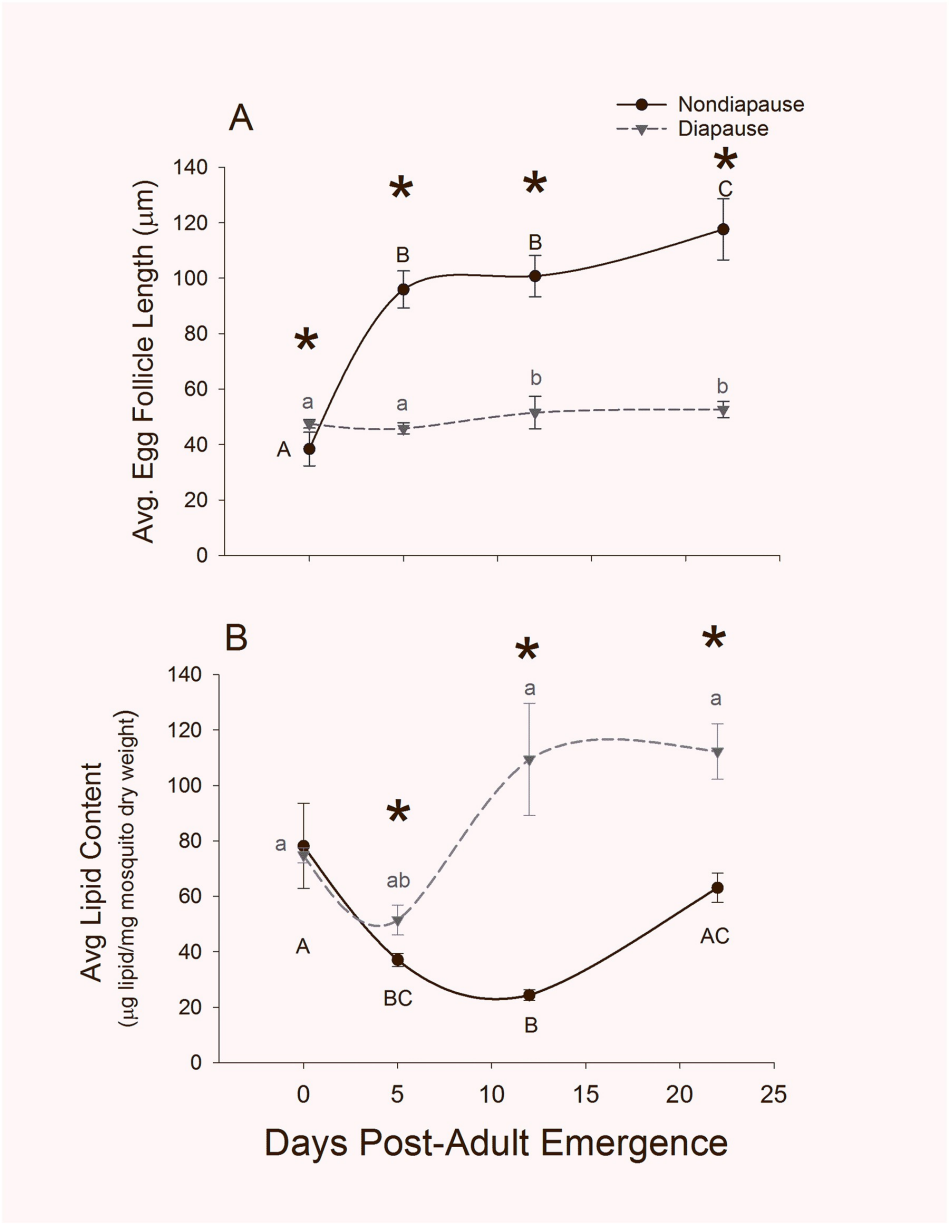
Changes in physical characteristics of diapausing and nondiapausing female mosquitoes. (A) Average egg follicle length of 12 females and (B) average lipid content in 4–5 females per time point and diapause status. Error bars represent s.e.m, and spline curves were fit to the data using SigmaPlot. Asterisks indicate significant differences in phenotypic markers between diapausing and nondiapausing females on the same day (Student’s T-test with Benjamini and Hochberg adjusted FDR, p < 0.05), while significant changes across the adult lifespan are represented with letters (black capital letters = nondiapausing mosquitoes; gray lowercase letters = diapausing mosquitoes; One-way ANOVA followed by Tukey’s Honest Significant Difference Test, p < 0.05).

Arrested ovarian development and lipid sequestration are hallmark features of diapause in *Cx*. *pipiens* [34, 35, 56]. Average egg follicle length (Fig 1A), a measure of ovarian development, was modestly, but significantly, higher in females programmed to enter diapause than in nondiapausing females on the day of adult emergence (mean ± s.e.m = 47.4 ± 1.5 μm for diapausing females and 38.4 ± 6.1 μm for nondiapausing females; FDR-adjusted p < 0.001). In nondiapausing females, follicle length increased significantly during the next 22 days (One way ANOVA, p < 0.001). The largest increase occurred during the first 5 days with a significant 2.5-fold increase in average length (Tukey’s post hoc comparison Day 0 to Day 5, p < 0.001), followed by a smaller but significant increase in egg follicle length from days 12 to 22 (~1.2-fold; Tukey’s post hoc comparison Day 12 to Day 22, p < 0.001). There was a slight but significant increase in the egg follicle lengths in diapausing females over the first 22 days of adult life (~1.1 fold increase between 0 and 22 d post adult emergence; One way ANOVA, p <0.001). However, nondiapausing females had significantly larger egg follicles than diapausing females on days 5, 12 and 22 egg (FDR-adjusted p < 0.001 for each time point).

There was no difference in average lipid content in pre-diapause females compared to females not programmed for diapause on the day of adult emergence (Fig 1B; FDR-adjusted p = 0.840). In nondiapausing females, there was a significant decrease in total lipid/mg dry weight over the next 22 d (One way ANOVA, p = 0.001) with total reduction of approximately 60% between 0 and 12 d (Tukey’s post-hoc comparison, p = 0.001). In diapausing females total lipid content increased ~1.5 fold during the 22 d following adult emergence. Diapausing females had significantly more total lipid than their nondiapausing counterparts every day except the day of emergence (Day 0, 5, 12 and 22 FDR-adjusted p = 0.839, 0.0179, 0.048 and 0.0179 respectively).

### Changes in miRNA abundance related to aging and diapause progression

**Fig 2:**
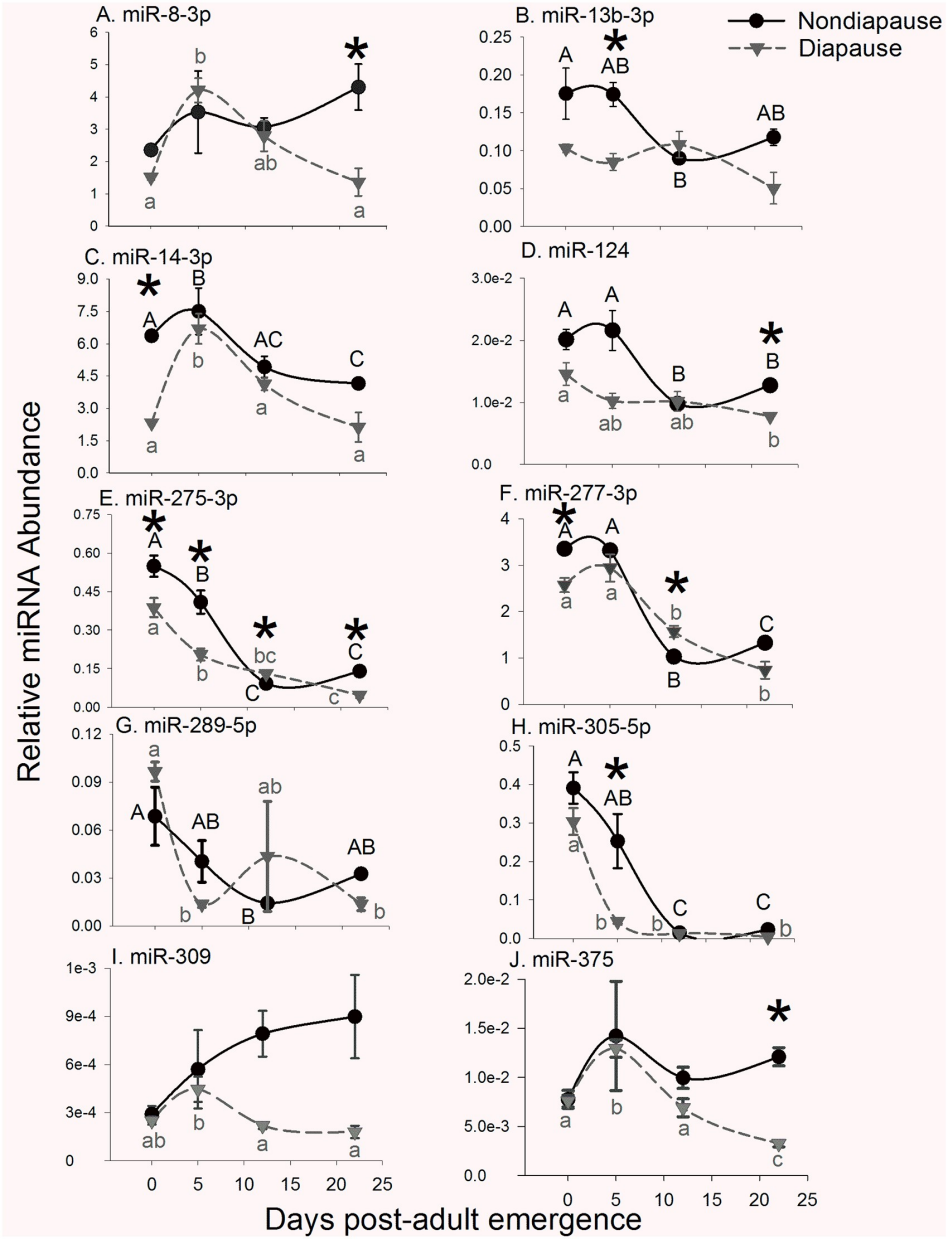
Profiles of candidate miRNAs nondiapausing and diapausing females during 22 d following adult emergence. Relative abundance of (A) miR-8-3p, (B) miR-13-3p, (C) miR-14-3p, (D) miR-124-3p, (E) miR275-3p, (F) miR-277-3p, (G) miR-289-5p, (H) miR-305-5p, (I) miR-309-5p and (J) miR-375-3p was measured with qRT-PCR. Each point represents the average relative miRNA abundance in 3–4 biological replicates each containing 3–5 whole female bodies. Error bars represent s.e.m. Data were normalized to the microRNA let-7, and spline curves were fit to the data using SigmaPlot. Asterisks indicate significant differences in miRNA abundance between diapausing and nondiapausing females (Student’s T-test with Benjamini and Hochberg adjusted FDR, p < 0.05), while different letters indicate significant changes in miRNA expression across adult lifespan (black capital letters = nondiapausing females; gray lowercase letters = diapausing females; one way ANOVA followed by Tukey’s Honest Significant Difference test, p < 0.05).

This set of experiments evaluated abundance of evolutionarily conserved, candidate miRNAs in diapause and nondiapausing adult females on the day of adult emergence (0 d) and on several additional days as adults aged (days 5, 12, and 22). MiRNAs evaluated include miR-8-3p, miR-13b-3p, miR-14-3p, miR-124-3p, miR-275-3p, miR-277-3p, miR-289-5p, miR-305-5p, miR-309-5p, and miR-375-3p. The abundance of all of these miRNAs dynamically and significantly changed during the 22 d evaluated. In general, miRNA abundance was higher on day 0 and lower on day 22 indicating a general decrease in abundance as adults aged (Fig 2).

In nondiapausing females there was an apparent ~ 2-fold increase in miR-8-3p (Fig 2A) abundance between 0 and 22 d post emergence that was not significant (One way ANOVA, p = 0.218). In diapausing females miR-8-3p abundance increased nearly 3-fold between 0 and 5 d followed by a decrease between days 5 and 22 (One Way ANOVA, p < 0.001; Tukey’s post-hoc comparison day 5 and day 22, p < 0.001). MiR-8-3p abundance was similar in diapausing and nondiapausing individuals except on days 0 and 22 when it was 1.5 and 3-fold more abundant in nondiapausing females than in diapausing females (Day 0 and 22 FDR-adjusted p = 0.0076 and 0.034, respectively).

There was a general decrease in the abundance of miR-13b-3p (Fig 2B) in nondiapausing mosquitoes (One way ANOVA, p = 0.029) but no significant change in diapausing mosquitoes over 22 d (One way ANOVA, p = 0.069). MiR-13b-3p was approximately 1.8-fold more abundant in nondiapausing mosquitoes than diapausing mosquitoes on days 0 and 5 (FDR-adjusted p = 0.16 and 0.043, respectively) but there was no significant difference on days 12 or 22 (FDR-adjusted p = 0.38 and 0.080 respectively).

MiR-14-3p (Fig 2C) significantly decreased ~1.5-fold in nondiapausing females (One-way ANOVA, p = 0.004). In diapausing females, there was a 3-fold increase between days 0 and 5 followed by a sharp decrease (One-way ANOVA, p < 0.001). On day 0, miR-14-3p was 4-fold more abundant in nondiapausing females compared to diapausing females (FDR-adjusted p < 0.001), but there were no significant differences between diapausing and nondiapausing females at any other time point.

MiR-124-3p (Fig 2D) abundance significantly decreased from 0 to 22 d in both nondiapausing (One way ANOVA, p < 0.001) between 5 and 12 d post-emergence and between 0 and 22 d in diapausing (One-way ANOVA, p = 0.028) females. It was significantly more abundant in nondiapausing females only on day 22 (FDR-adjusted p <0.001). Although miR-124 appeared to be upregulated in nondiapausing females on days 0 and 5 (1.38 and 2-fold, respectively), these differences were not significant (FDR-adjusted p = 0.08 and 0.08 respectively).

MiR-275-3p (Fig 2E) decreased significantly in both nondiapausing (One-way ANOVA, p < 0.001) and diapausing females (One-way ANOVA, p < 0.001). In nondiapausing females, miR-275-3p decreased ~80% between days 0 and 12, and then remained unchanged between 12 and 22 d. In diapausing females there was a 5-fold decrease between 0 and 12 d; but no significant change between 12 and 22 d. MiR-275-3p was up to 2-fold more abundant nondiapausing females compared to diapausing females on days 0, 5, and 22 (FDR-adjusted p = 0.028, 0.028 and 0.002, respectively). MiR-275-3p was also a statistically more abundant in diapausing females than nondiapausing females on day 12 (FDR-adjusted p = 0.01).

Similarly, the abundance of miR-277-3p, miR-289-5p and miR-305-5p significantly decreased as females aged. Specifically, miR-277-3p (Fig 2F) decreased 2.5 fold in nondiapausing females (One way ANOVA, p < 0.001) and 3.5 fold in diapausing females (One way ANOVA, p <0.001) from day 0 to 22. MiR-277-3p was underexpressed in diapausing females on day 0 (FDR-adjusted p = 0.027) but was not significantly different from nondiapausing females at other times. MiR-289-5p (Fig 2G) abundance significantly decreased over time in nondiapausing (One way ANOVA, p = 0.028) and diapausing females (One Way ANOVA, p = 0.018) but there were no significant differences in miR-289-5p abundance between diapausing and nondiapausing females. Between day 0 and day 22 miR-305-5p (Fig 2H) decreased 17-fold in nondiapausing females (One way ANOVA, p < 0.001) and 80-fold in diapausing females (One way ANOVA, p < 0.001). MiR-305-5p was underexpressed in diapausing females, relative to nondiapausing females, on day 5 post-emergence (FDR-adjusted p = 0.013), but not at other time points.

MiR-309-5p abundance (Fig 2I) did not significantly change in nondiapausing females during the 22 d following adult emergence (One way ANOVA, p = 0.147), but increased significantly in diapausing females on day 5 followed by a decrease to its initial level on days 12 and 22 (One way ANOVA, p = 0.008). Beginning on day 12, there was an apparent 3-fold difference between diapausing and nondiapausing females that was due to a substantial increase in miR-309-5p abundance that was not observed in diapausing females. However, this difference was not significant (FDR-adjusted p-values = 0.104 and 0.137 on days 12 and 22 respectively).

MiR-375-3p (Fig 2J) abundance did not change significantly between 0 and 22 d following adult emergence in nondiapausing females (One way ANOVA, p = 0.306), but in diapausing females there was a significant increase in miR-375-3p between 0 and 5 d followed by significant decrease on days 12 and 22 (One Way ANOVA, p < 0.001). MiR-375-3p was ~3.7-fold more abundant in nondiapausing females than in diapausing females 22 d post-adult emergence (FDR-adjusted p = 0.004).

### Target identification of diapause relevant miRNAs

The functional significance of differentially regulated miRNAs depends on the identity of the genes they regulate. Identifying gene targets of an individual miRNA is complicated because a single miRNA can regulate multiple mRNAs and a single mRNA can be regulated by multiple miRNAs. To maximize our ability to identify diapause relevant targets of miRNAs, we used TargetScan Fly, release 6.2 [57–59], to identify miRNAs that may regulate transcripts of candidate genes that are known, from previously published studies, to be differentially regulated in diapausing *Cx*. *pipiens* [8, 41]. We also used DIANA mirPath 3.0 [59] to identify KEGG pathways that may be regulated by miRNAs that were differentially regulated 0 and 5 d post-adult emergence. These times were selected because the largest differences in miRNA abundance between diapausing and nondiapausing females was seen at these points and because of the timing relative to observed changes in egg follicle size and total lipid content.

Multiple studies have identified numerous genes that are differentially regulated in diapausing females of *Cx*. *pipiens*, many of which are putative targets of miRNAs evaluated in this study. Fifteen of the thirty-three genes that regulate fat metabolism in diapausing females of *Cx*. *pipiens* are predicted targets of miRNAs [39] (S2 Table). MiR-277-3p potentially regulates eleven genes including *acc* and *fabp*, which encode Acetyl-CoA carboxylase and Fatty acid binding protein, respectively. In addition, *fad-2* and *fad-3*, two genes which both encode Δ(9)-desaturase enzymes, are putative targets of miR-305-5p (*fad-2*) or miR-8-3p and miR-124-3p (*fad-3*).

A meta-analysis of diapause-relevant genes in insects [60] identified 572 *Drosophila* transcripts that are orthologous to diapause-relevant genes in *Cx*. *pipiens* [8]. Of these, 138 are putative targets of the miRNAs evaluated in this study. These include 39 putative targets of miR-277-3p, 21 targets of miR-375-3p, and 15 targets of miR-13b-3p (S2 Table). Most of these putative targets were not differentially regulated in diapausing *Cx*. *pipiens* [8, 60]. However, miRNAs can repress translation without degrading the transcript [61], and it is possible for a gene to be regulated by a miRNA without a significant change in transcript abundance.

DIANA mirPath 3.0 was used to identify KEGG pathways that have genes that are regulated by specific miRNAs. We identified 8 KEGG pathways, including Mucin type O-Glycan biosynthesis; Valine, leucine and isoleucine degradation; Fatty acid elongation; Fatty acid degradation; Propanoate metabolism; MAPK signaling pathway; Hippo signaling pathway; and Valine, leucine and isoleucine biosynthesis, that may be regulated collectively by miR-14-3p, miR-275-3p, miR-13b-3p, miR-124-3p, miR-277-3p, and miR-305-5p (Table 1). It is important to note that all of these microRNAs were significantly lower in diapausing females 0 and/or 5 d post-adult emergence, suggesting that these pathways are regulated as females prepare to enter diapause.

**Table 1:** KEGG pathways that may include genes regulated by miRNAs that are differentially regulated before diapause entry*.

KEGG pathway	p-value	Genes		miRNAs
		*D*. *melanogaster* gene	*Cx*. *quinquefasciatus* ortholog**	
Mucin type O-Glycan biosynthesis	1.53E-25	CG30463	CPIJ005695	miR-124, miR-277
		pgant3	CPIJ009408	miR-13b
		GalNAc-T1	CPIJ011238 CPIJ015084	miR-277
		pgant5	CPIJ007696	miR-277
		pgant2	CPIJ009181	miR-14
		Pgant35A	CPIJ017883	miR-14
Valine, leucine and isoleucine degradation	1.38E-19	scu	CPIJ014285	miR-277
		CG5599	CPIJ006326	miR-277
		CG8199	CPIJ011779	miR-277
		CG15093	CPIJ015209	miR-277
		CG3902	CPIJ009148	miR-277
		CG2118	CPIJ003841	miR-277
		CG5044	CPIJ012030	miR-277
		yip2	CPIJ002342	miR-277
		CG6638	CPIJ014783	miR-277
		CG4860	CPIJ011633	miR-277
		CG1673	CPIJ015408	miR-277
		CG6543	CPIJ006455 CPIJ014621	miR-277
		CG8778	CPIJ016903 CPIJ006767 CPIJ019824	miR-277
		CG3267	CPIJ009999	miR-277
		CG10932	CPIJ019232	miR-277
		CG12262	CPIJ008217	miR-305
		CG30463	CPIJ005695	miR-124
Fatty acid elongation	8.03E-09	yip2	CPIJ002342	miR-277
		CG6543	CPIJ006455 CPIJ014621	miR-277
		CG16935	CPIJ014619 CPIJ006453	miR-13b
Fatty acid degradation	3.52E-06	CG3902	CPIJ009148	miR-277
		yip2	CPIJ002342	miR-277
		CG4860	CPIJ011633	miR-277
		CG6543	CPIJ006455 CPIJ014621	miR-277
		CG9547	CPIJ000375	miR-277
		CG10932	CPIJ019232	miR-277
		CG12262	CPIJ008217	miR-305-5p
Propanoate metabolism	4.51E-06	CG17896	CPIJ009984	miR-277
		CG5044	CPIJ012030	miR-277
		CG6543	CPIJ006455 CPIJ014621	miR-277
		ACC	CPIJ005524	miR-277
		CG10932	CPIJ019232	miR-277
		Sucb	CPIJ005779	miR-277
		AcCoAS	CPIJ016639	miR-13b
		CG12262	CPIJ008217	miR-305-5p
MAPK signaling pathway—fly	0.001802	Egfr	CPIJ009984	miR-277 miR-124
		phl	CPIJ004499	miR-277
		Gap1	CPIJ015188	miR-277
		drk	CPIJ000806	miR-277
		tsl	CPIJ013682	miR-13b
		tll	CPIJ012467	miR-13b miR-124
Hippo signaling pathway—fly	0.003748	ex	CPIJ000761	miR-277
		Rassf	CPIJ010185 CPIJ010183	miR-277
		Act42A	CPIJ016462 CPIJ009808 CPIJ005785 CPIJ012573	miR-277
		dco	CPIJ003503	miR-277
		14-3-3zeta	CPIJ008589	miR-277
		ds	CPIJ017647 CPIJ001491 CPIJ001492	miR-277
		upd3	No non-Drosophilid orthologies	miR-13b miR-305-5p
		upd2	No non-Drosophilid orthologies	miR-13b
		fj	CPIJ001724	miR-13b
		Mer	CPIJ019642 CPIJ007590	miR-124
		kibra	CPIJ018899	miR-124
		sav	CPIJ006349	miR-124
Valine, leucine and isoleucine biosynthesis	0.026761	CG1673	CPIJ015408	miR-277

*DIANA mirPath v3.0 query of microT-CDS using miR-14-3p, miR-275-3p, miR-13b-3p, miR-124-3p, miR-277-3p, miR-305-5p

** *Cx*. *quinquefasciatus* orthologs identified via OrthoDB v9.1 (EOG091502GM)

### Changes in miRNA abundance related to blood feeding

**Fig 3:**
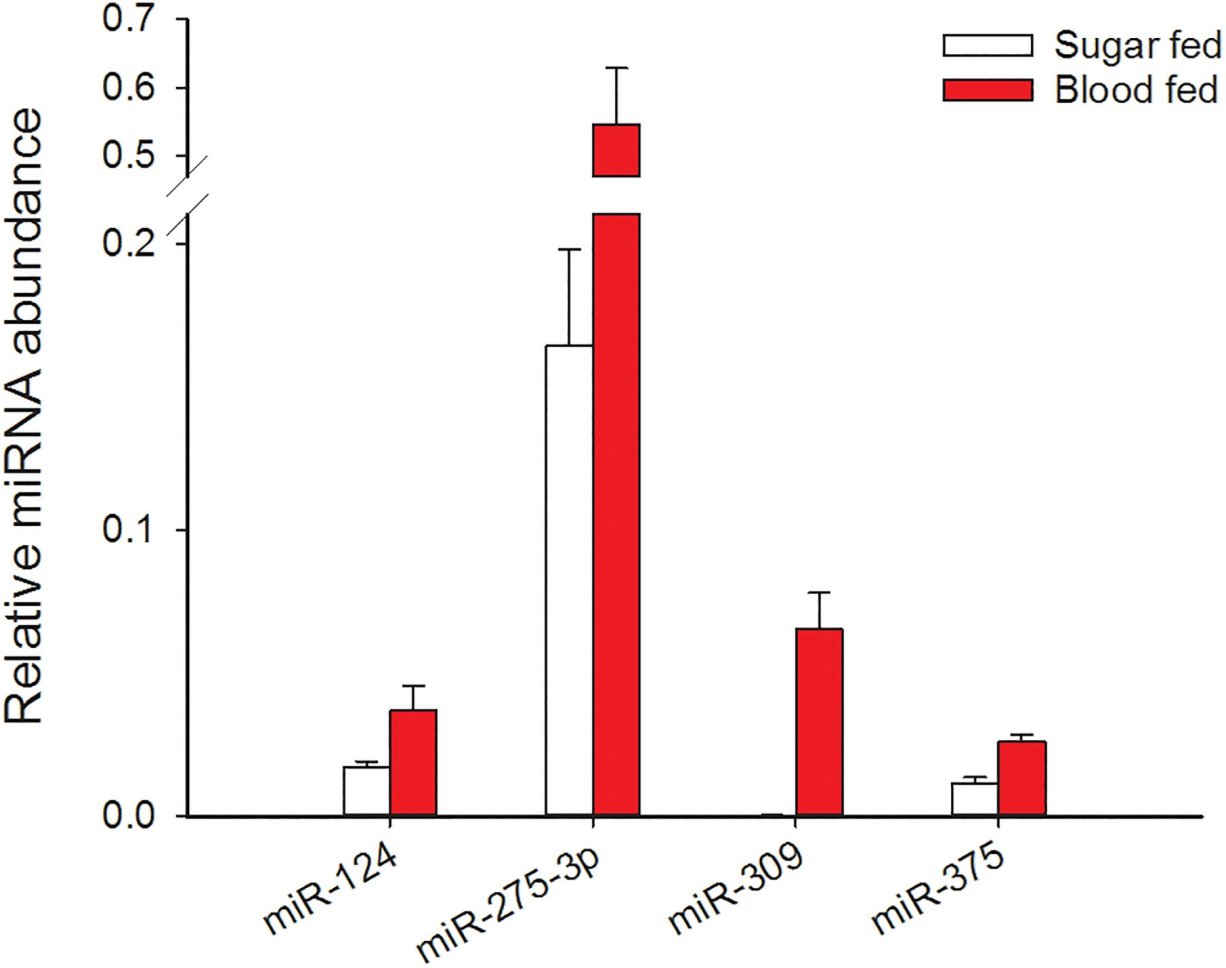
Several miRNAs are upregulated following a blood meal. Relative miRNA abundance was measured with qRT-PCR. Bars represent the mean ± s.e.m of in 4 independent, biological replicates each containing 3–5 whole body, nondiapausing females. All females were 12 days old and blood fed females ingested a blood meal 36 h before collection. Blood feeding did not significantly change the abundance of miR-124-3p (FDR-adjusted p = 0.102) but did significantly increase the abundance of miR-275-3p, miR-309-5p and miR-375-3p (FDR-adjusted p < 0.05).

This set of experiments evaluated changes in abundance of candidate miRNAs known to be differentially regulated following a blood meal in *Aedes aegypti* and *Anopheles stephensi* mosquitoes [45, 46] but have not previously been examined in female *Cx*. *pipiens* mosquitoes. MiR-124-3p, miR275-3p, miR-309-5p, and miR-375-3p were measured in long-day reared, nondiapausing females 12 days after adult emergence that had never received a blood meal (sugar-fed control) or had been given a blood meal 36 h prior to sampling (Fig 3). There was no significant change in miR-124-3p abundance (FDR-adjusted p = 0.102) but miR-275-3p, miR-309-5p and miR-375-3p were all significantly upregulated in blood-fed females (FDR-adjusted p = 0.021 for each).

## Discussion

Diapause is a complex phenotype that depends on coordinated regulation of multi-gene networks that are themselves regulated by numerous factors, including microRNAs. The results of this study indicate that multiple microRNAs are differentially regulated in pre-diapause and diapausing females of *Cx*. *pipiens* mosquitoes compared to their nondiapausing counterparts. Specifically, miR-8-3p, miR-13b-3p, miR-14-3p, miR-275-3p, and miR-305-5p were underexpressed in diapause-destined females on days 0 and 5 following adult emergence and likely regulate diapause entry and/or the switch from a developmental pathway that lacks diapause to one that includes period of dormancy. In addition, miR-8-3p, miR-124, miR-275-3p and miR-375-3p were underexpressed in diapausing females 22 d post-emergence and may be important for maintaining the diapause phenotype.

Our results show clear differences in the abundance of numerous miRNAs in diapausing females compared to their nondiapausing counterparts. However, the biological relevance of these differences is difficult to interpret because the influence a particular miRNA has on such a complex phenotype depends on both the abundance of the miRNA and the number of target sites present in a given cell or tissue, and whether it positively or negatively regulates a gene target. All of the miRNAs evaluated in this study were underexpressed in pre-diapause or diapausing females compared to their continuously developing (i.e. nondiapause) counterparts which suggests that during diapause, reduced miRNA abundance allows genes that are repressed in nondiapausing females to be turned on. However, diapause is typically characterized by wide-spread downregulation of gene expression [1], and, thus, the role of miRNAs in diapausing insects is likely more nuanced than this interpretation suggests.

The functional significance of these changes in miRNA abundance, and how they regulate diapause also depends on the function of the genes they target. There is currently limited information about how specific miRNAs regulate gene expression in any mosquito species. In particular, the targets and functions of miRNAs in *Cx*. *pipiens* remain to be tested. However, some inferences can be made based on computationally predicted gene targets and from studies on other insects including *D*. *melanogaster* and mosquitoes including *Aedes aegypti* and *Anopheles stephensi*. To facilitate the discussion, we focus on miRNAs that have known roles in circadian timing, lipid metabolism, and ovarian development, which are key processes in the establishment and maintenance of diapause in *Cx*. *pipiens* [34, 39, 43].

Diapause in *Cx*. *pipiens* is associated with changes in the expression profiles of core components of the circadian clock [43]; and miR-124-3p, expressed primarily in the brains of mosquitoes [62], may regulate some of these changes. In *D*. *melanogaster*, miR-124-3p regulates circadian output by targeting the gene encoding the CLOCK protein [63–65]. MiR-124-3p also mediates daily phases of locomotor activity by regulating genes in the Bone Morphogenetic Protein signaling pathway [66, 67]. Therefore, we hypothesize that miR-124-3p regulates behavioral and physiological outputs, such as changes in sugar feeding activity [68], that are downstream of the circadian clock in diapausing *Cx*. *pipiens*. It is important to note, however, there are some fundamental differences in the regulation of the circadian clock in *Cx*. *pipiens* compared to *D*. *melanogaster* [69], and additional studies are needed to confirm the link between miRNAs, circadian rhythms, and diapause in *Cx*. *pipiens*.

Diapause in *Cx*. *pipiens* is characterized by fat accumulation, through overall suppression of insulin signaling [40, 70]. We found that, on the day of adult emergence (i.e., day 0) there was no difference in the total lipid content of short-day reared, diapause-destined females compared to nondiapause-destined females. However, there was a significant, 1.5 fold (FDR-adjusted p = 0.006) difference between the two groups by day 5, indicating rapid fat accumulation occurs in diapausing females during this time. Indeed, this is consistent with previously reported changes lipid accumulation in diapausing females [35, 56] and is also consistent with changes in mRNA expression for several genes associated with fat metabolism 1 week after adult eclosion [38]. Three miRNAs, miR-14-3p, miR-277-3p, and miR-305-5p that are known to regulate fat metabolism and/or insulin signaling [71–74], were downregulated in diapause-destined mosquitoes during this time, and, thus, are thought to contribute to the observed changes in lipid metabolism in mosquitoes entering diapause.

MiR-277-3p regulates insulin signaling directly by targeting genes that encode Insulin-like peptides (Ilps) [75] and indirectly by regulating branched-chain amino acid (i.e. valine, leucine, and isoleucine) metabolism [72]. In *Ae*. *aegypti* mosquitoes, knockdown of miR-277-3p reduces lipid storage in the fat body by upregulating insulin signaling and promoting nuclear export of the FOXO transcription factor [75, 76]. In *D*. *melanogaster*, miR-277-3p indirectly mediates insulin signaling through regulation of branched-chain amino acid (BCAA) metabolism [72]. BCAAs activate TOR signaling and insulin secretion and regulate lifespan in evolutionarily diverse species [72, 77, 78]. Indeed, valine, leucine and isoleucine biosynthesis was one of the KEGG pathways that our computational analyses predicted to be regulated by miR-277-3p and other microRNAs that we observed were downregulated early in diapause. Taken together, these studies in *Ae*. *aegypti* and *D*. *melanogaster* and our results suggest that miR-277-3p regulates multiple aspects of insulin signaling and fat metabolism during diapause in *Cx*. *pipiens* and may be a critical player in the generation of the fat hypertrophy that is a hallmark of diapause in this species.

Our observation that miR-305-5p decreases dramatically as *Cx*. *pipiens females age* is consistent with changes in miR-305 abundance in aging adults of *D melanogaster*. In *D*. *melanogaster*, miR-305-5p regulates lifespan by targeting members of the insulin signaling pathway including *dilp6* and *dilp8* [73, 74], and miR-305 may targets these genes in *Cx*. *pipiens* as well. The decrease in miR-305 occurs sooner in diapausing females, which suggests an additional, diapause-specific function. MiR-305 also suppresses *p53* in fat body cells in *D*. *melanogaster* [26]. Decreased miR-305 increases *p53* and promotes lipid storage and improves resistance to starvation in *D*. *melanogaster* could contribute to the increase in total lipids observed in diapause *Cx*. *pipiens*.

MiR-305-5p and miR-277-3p may also mediate lipid accumulation during diapause in *Cx*. *pipiens* by regulating additional genes that belong to the Fatty acid elongation and Fatty acid degradation pathways. Six genes known to be differentially regulated in diapausing females of *Cx*. *pipiens* 7 days post-emergence [38] are computationally predicted targets of miR-305-5p and miR-277-3p. One gene of particular interest is *acd-1*, a putative target of miR-305-5p, which encodes an Acyl-CoA dehydrogenase and is overexpressed by ~2-fold in diapausing females 7 d post-emergence [38]. Suppression of miR-305-5p on day 5 is consistent with upregulation of *acd-1* two days later. However, the putative interaction of miR-305-5p and *acd-1* is based on the sequence of this gene in *D*. *melanogaster*, and additional experiments are needed to confirm that miR-305-5p regulates *acd-1* in *Cx*. *pipiens*.

In *D*. *melanogaster*, miR-14-3p suppresses insulin signaling by inhibiting its target *sugarbabe*, such that low levels of miR-14 lead to an increase in the level of insulin-like peptides [79]. Mutant flies lacking miR-14-3p store fat while flies overexpressing miR-14-3p are lean [71, 79]. Lower levels of miR-14-3p on day 0 in diapause-destined flies could indicate that miR-14-3p also regulates fat metabolism in *Cx*. *pipiens*. However, it is difficult to rationalize the changes in miR-14-3p abundance in diapausing and nondiapausing females, as insulin signaling is suppressed in diapausing females [41] while lipid content is elevated (Fig 1). In addition, it is not clear whether the function of miR-14-3p in *D*. *melanogaster* is conserved in other insects. The 3’ UTR sequence of *sugarbabe* that is available for *Cx*. *quinquefasciatus* (CPIJ007837) does not appear to have a region that miR-14-3p can bind. However, there are multiple regions within the open reading frame (ORF) of *sugarbabe* where miR-14-3p could potentially bind. It will be interesting to see whether the interaction between miR-14-3p that occurs in *D*. *melanogaster* is conserved in mosquitoes and whether miR-14-3p regulates insulin signaling and fat metabolism in diapausing females of *Cx*. *pipiens*.

Arrested ovarian development is also a key feature of the diapause phenotype in *Cx*. *pipiens* [34]. Surprisingly, diapause-destined females had significantly larger egg follicles than nondiapausing females on the day of adult emergence (i.e. day 0). A significant increase in egg follicle length in nondiapausing females, but not in diapausing females, between days 0 and 5 suggests that ovarian development is suppressed in diapausing females during this time. Numerous miRNAs have experimentally validated roles in ovarian development in other mosquito species, including miR-8-3p, miR-275-3p, miR-309-3p, and miR-375-3p [44, 45, 80–82]. Of these, only miR-8-3p and miR-275-3p were differentially expressed early in the adult stage, prior to diapause entry. MiR-8-3p, miR-275-3p, and miR-375-3p were all significantly underexpressed in diapausing females on day 22 post-emergence. Together these data suggest the absence of at least some miRNAs is important for continuously arresting egg follicle development and other pathways that maintain diapause in *Cx*. *pipiens*.

Mosquito species from multiple genera require a blood meal for ovarian development [reviewed in 82] and in at least two species, *Aedes aegypti* and *Anopheles stephensi*, miR-275-3p, miR-309-3p, and miR-375-3p are upregulated following a blood meal [43, 46, 79–81, 83]. To evaluate whether the regulation and, possibly, function of these miRNAs is also conserved in *Culex* mosquitoes, we measured changes in the abundance of miRNA in nondiapausing females following a blood meal. Indeed, we found that increases in the abundance of these miRNAs were stimulated by blood feeding in *Cx*. *pipiens*. However, there are significant differences in the baseline levels of these miRNAs, and in egg follicle length, between diapausing and nondiapausing females in the absence of a blood meal (Fig 1A). Therefore miR-275-3p, miR-309-3p, and miR-375-3p are likely suppressed as part of the diapause program, independent of blood feeding.

**Fig 4:**
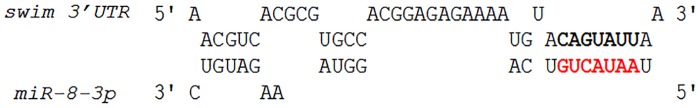
Predicted interactions between miR-8-3p and its putative target *secreted wingless-interacting molecule (swim)*. The interaction between the seed sequence of miR-8-3p (in red) and the complementary sequence in the 3’ UTR of *swim*, indicated with bold text, is energetically favorable (MFE = -21.56).

Of the miRNAs that regulate ovarian development, miR-8-3p is of particular interest because it also targets genes involved in fat metabolism. In *D*. *melanogaster*, miR-8-3p regulates insulin signaling by inhibiting translation of U-shaped, a protein that activates phosphatidylinositol 3-kinase (PI3K) [84]. The 3’ UTR is not available for the *Cx*. *pipiens* ortholog of U-shaped, so it is not known whether miR-8-3p regulates this gene in this mosquito species. In *Ae*. *aegypti* miR-8-3p is expressed in the fat body and coordinately regulates both fat metabolism and reproduction by targeting *secreted wingless-interacting molecule* (*swim*), a gene in the Wingless signaling pathway [80]. The *Culex* ortholog of *swim* (CPIJ008716) contains a region in the 3’ UTR where miR-8-3p can bind (Fig 4), thus it is likely this miR-8-3p function observed in *Ae*. *aegypti* is conserved in *Culex* mosquitoes. It will be interesting to further investigate how miR-8-3p regulates adult reproductive diapause in *Cx*. *pipiens*.

Many of the miRNAs that we found to be differentially regulated in diapausing *Cx*. *pipiens* are also differentially regulated in diapausing pupae of *S*. *bullata* [30] indicating that microRNAs could be considered part of a “toolkit” of mechanisms that mediate diapause entry and diapause maintenance in insects. Specifically, miR-13b-3p, miR-275-3p, and miR-305-5p were underexpressed in diapausing pupae compared to their nondiapause counterparts [30]. It is difficult to directly compare changes in miRNA abundance between these two species because of difference in the life stages when they enter diapause. However, in both *S*. *bullata* and *Cx*. *pipiens* these miRNAs were also underexpressed during diapause, which indicates these microRNAs may have similar roles in the regulation of diapause in diverse insects.

Taken together, our results provide compelling evidence that miRNAs are part of the toolkit of molecular mechanisms that regulate diapause in female adults of *Cx*. *pipiens* mosquitoes. We identified a number of microRNAs that were differentially regulated in diapause-destined females compared to females not programmed for diapause, on the day of adult emergence, which suggests they are important for initiating changes in ovarian development and lipid abundance that become apparent several days later. Overall, we found the abundance of microRNAs evaluated in this study was reduced in pre-diapause and diapausing females compared to their nondiapause counter-part, and thus it may be the absence of these miRNAs that promotes physical changes associated with diapause entry. However, miRNA expression in adult female *Cx*. *pipiens* is dynamic and changes over time regardless of diapause status. The observation that miRNA abundance fluctuates even in diapausing females indicates that diapause is not simply a state of suspended animation but is a dynamic alternative developmental pathway. In addition, the complex nature of miRNA gene regulation, with each miRNA having the potential to either positively or negatively regulate hundreds of genes, suggest countless possibilities in how the differences in miRNA abundance gene expression. Our *in silico* analyses have uncovered several diapause-relevant genes and pathways that are likely regulated by the microRNAs examined in this study. However, the functional relevance of these miRNAs needs to be experimentally validated. Combined with previous studies on other diapausing insects including flesh flies and moths, it appears that microRNA regulation of diapause is wide-spread in evolutionarily diverse insect species.

## Supporting information

S1 Table Sequence of each miRNA-specific primer and its efficiency (E) and regression coefficient (R^2^) from standard curve analyses. (DOCX)

S2 Table Putative miRNA target genes involved in fat metabolism as identified by Sim and Denlinger, 2009. (XLSX)

S3 Table Putative miRNA targets in diapausing *Cx*. *pipiens*. Adapted from data collected by Kang et al., 2015 and analyzed by Ragland and Keep, 2017.
(XLSX)

S1 Fig Consistent cycle threshold (C_t_) values for let-7 demonstrate that it is a viable reference gene. (A) Abundance of let-7 in diapausing and nondiapausing females collected on days 0, 5, 12 and 22 (n = 31); (B) abundance of let -7 sugar fed and blood fed nondiapausing female mosquitoes (n = 8).
(TIF)
