# Supplementary material for: Evidence that microRNAs are part of the molecular toolkit regulating adult reproductive diapause in the mosquito, Culex pipiens
Source: PLoS One. 2018 Nov 29;13(11):e0203015. doi: 10.1371/journal.pone.0203015 (PMC6264513; doi:10.1371/journal.pone.0203015)
Supplement: S1 Table — (DOCX) [file pone.0203015.s001.docx]

S1 Table Primer sequences used for qRT-PCR

| **miRNA** | **Sequence** | **Efficiency (%)** | **R^2^** |
| --- | --- | --- | --- |
| cqu-let-7-5p | UGAGGUAGUUGGUUGUAUAGU | 103.8 | 0.983 |
| cqu-miR-8-3p | UAAUACUGUCAGGUAAAGAUGUC | 90.8 | 0.993 |
| cqu-miR-13b-3p | UAUCACAGCCAUUUUGACGAGUU | 87 | 0.995 |
| cqu-miR-14-3p | UCAGUCUUUUUCUCUCUCCUAU | 91.5 | 0.990 |
| cqu-miR-124-3p | UAAGGCACGCGGUGAAUGC | 96.4 | 0.997 |
| cqu-miR-275-3p | UCAGGUACCUGAAGUAGCGCGCG | 99.9 | 0.991 |
| cqu-miR-277-3p | UAAAUGCACUAUCUGGUACGACA | 89 | 0.966 |
| dme-miR-289-5p | UAAAUAUUUAAGUGGAGCCUGCGACU | 89.4 | 0.991 |
| cqu-miR-305-5p | AUUGUACUUCAUCAGGUGCUCUGG | 89.5 | 0.993 |
| cqumiR-309-3p | UCACUGGGCAUAGUUUGUCGC | 102.5 | 0.991 |
| cqu-miR-375-3p | UUUGUUCGUUUGGCUCGAGUUA | 100.7 | 0.987 |
