# Supplementary figures and images for: Evidence that microRNAs are part of the molecular toolkit regulating adult reproductive diapause in the mosquito, Culex pipiens
Source: PLoS One. 2018 Nov 29;13(11):e0203015. doi: 10.1371/journal.pone.0203015 (PMC6264513; doi:10.1371/journal.pone.0203015)

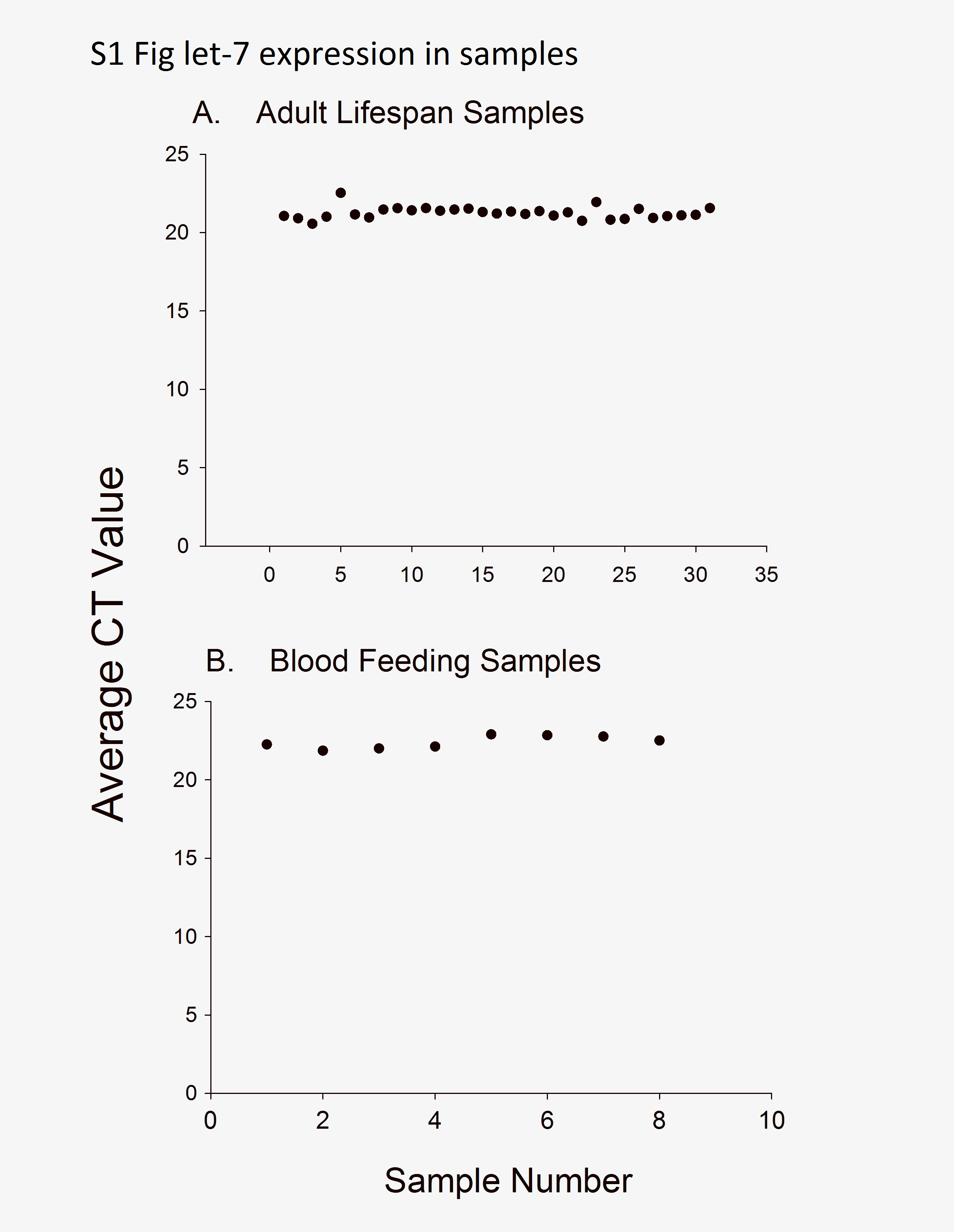

Supplement: S1 Fig — (A) Abundance of let-7 in diapausing and nondiapausing females collected on days 0, 5, 12 and 22 (n = 31); (B) abundance of let -7 sugar fed and blood fed nondiapausing female mosquitoes (n = 8). (TIF) [file pone.0203015.s004.tif]
